# Factors Associated With Nutritional Status in Patients With Removable Dentures: A Cross-Sectional Study

**DOI:** 10.7759/cureus.75288

**Published:** 2024-12-07

**Authors:** Ranko Kawata, Yuka Abe, Yuriko Kusumoto, Takashi Matsumoto, Takumi Yokoi, Haruka Sako, Kazuyoshi Baba

**Affiliations:** 1 Department of Prosthodontics, Graduate School of Dentistry, Showa University, Tokyo, JPN; 2 Division of Prosthodontics, Department of Prosthodontics, School of Dentistry, Showa University, Tokyo, JPN

**Keywords:** controlling nutritional status, malnutrition, masticatory function, nutritional status, removable dentures, undernutrition

## Abstract

Background

Tooth loss can impair masticatory function and may subsequently result in malnutrition. This study aimed to investigate the factors associated with controlling nutritional status (CONUT) scores, which assess malnutrition risk, in patients with removable dentures.

Materials and methods

In this cross-sectional study, data were obtained from a consecutive sampling of 192 patients (mean age 72.7 ± 8.4 years, 73 males and 119 females) with removable dentures. Variables assessed included age, sex, body mass index (BMI), Eichner index, Charlson comorbidity index (CCI), social background (frequency of nutrition-related media consumption, level of attention to diet, economic status, educational background, and whether the patient lived alone), denture quality assessed by dentists, the Oral Health Impact Profile (OHIP), masticatory performance score based on the glucose concentration of comminuted gummy jelly, energy intake based on the brief-type self-administered diet history questionnaire (BHDQ), and the CONUT score as determined from blood test results. Multiple regression analysis was performed with malnutrition risk level (CONUT 0 as low-risk, CONUT 1 and 2 as mild-risk, and CONUT 3 and 4 as moderate-risk) as the dependent variable and the 14 items aforementioned as independent variables.

Results

The number of patients at low, mild, and moderate risk was 67, 116, and 9, respectively. Regression analysis revealed that being male (*P* = 0.004), low BMI (*P* < 0.001), and low masticatory performance scores (*P* = 0.025) were significantly associated with malnutrition risk, as evaluated by the CONUT score.

Conclusion

Enhanced masticatory performance may help improve the nutritional status of patients with removable dentures.

## Introduction

The primary role of prosthetic dentistry is to restore masticatory function impaired by tooth loss [1]. Studies have shown that masticatory function and oral health-related quality of life (OHRQoL) [2] can be improved by prosthetic treatments [3].

Impaired masticatory function due to missing teeth may lead to changes in dietary habits, resulting in deficiencies in certain nutrients [4], frailty [5], and psychosocial consequences [6]. Prosthetic treatment with removable dentures can improve nutritional status as evaluated by the Mini Nutritional Assessment (MNA) and body mass index (BMI) [7,8]. Additionally, masticatory performance - defined as a measure of the ability to comminute food under standardized test conditions - has been reported to be significantly associated with food and nutrient intake as assessed by the food frequency questionnaire and the brief-type self-administered diet history questionnaire (BDHQ) [9,10]. These findings suggest that improved masticatory function achieved by prosthetic treatment may improve dietary habits, potentially leading to improved nutritional status; however, it has not been demonstrated.

Currently, the Global Leadership Initiative on Malnutrition (GLIM) criteria, which integrate a nutritional screening tool with malnutrition diagnosis based on phenotype and etiology, are recommended for assessing nutritional status in adults [11]. Nevertheless, assessments using screening tools such as MNA may cause over- or underestimation of changes in dietary intake because of self-reported information. Blood tests are routinely used in clinical practice because they can objectively and quickly assess nutritional deficiencies and general conditions. Clinical studies indicate that the number of remaining teeth [12] and masticatory performance [9] are significantly associated with serum albumin levels. Although serum albumin levels by blood test have traditionally been considered an indicator of nutritional status, they can be influenced by factors such as inflammation [13], which limits the interpretation of nutritional status that relies on serum albumin levels alone. The controlling nutritional status (CONUT) comprehensively assesses serum albumin, total cholesterol, and lymphocyte count rather than relying on a single parameter, making it a proposed objective tool for nutritional assessment [14].

As a first step in demonstrating that improved masticatory function by prosthetic treatment can contribute to the improvement of nutritional status, this study aimed to investigate factors and their relative influence on objective nutritional status, as evaluated by the CONUT score for malnutrition risk, in university clinic outpatients wearing removable dentures. The null hypothesis of this study was that no factors are associated with the malnutrition risk levels, as determined by the CONUT score, in this population.

## Materials and methods

Study population

In this cross-sectional study, participants were consecutively sampled at a university dental hospital (Tokyo, Japan) from August 2023 to July 2024. Patients using removable dentures for at least one month were included. Exclusion criteria included individuals who had received dietary control within the past six months; those residing in or attending a nursing home; individuals with a history of malignant tumors; the presence of renal failure, nephrotic syndrome, hepatic failure, cirrhosis, or acute inflammatory conditions; individuals unable to comprehend or respond appropriately to self-administered questionnaires; and those wearing implant overdentures.

Sample size calculation

According to multiple regression analysis guidelines, which recommend a sample size of at least 50 + 8 × (number of predictors) [15], a minimum of 162 participants was required for a model with 14 independent variables to ensure adequate power and reliable estimates. To account for potential dropout, the sample size was increased to 200.

Data collection

BMI was calculated using self-reported height and weight. The Charlson comorbidity index (CCI) was employed to assess comorbidities [16]. In this study, CCI results were categorized into two groups: CCI = 0 and CCI ≥ 1, considering the study population with relatively few comorbidities.

An original self-administered questionnaire was utilized to investigate social backgrounds. Participants were asked to respond to the following questions on a four-point Likert scale: “How often do you check TV programs, magazines, the internet, or other sources related to health and nutrition?” (nutrition-related media consumption); “To what extent do you pay attention to maintaining a healthy diet?” (attention to a healthy diet); and “To what extent do you currently feel financially comfortable?” (financial comfort). The responses were categorized as either “high” or “low.” Participants were also asked to provide information about their highest level of education (high school or above) and living arrangements (whether they lived alone).

OHRQoL was assessed using the Japanese version of the Oral Health Impact Profile (OHIP-J) [17], which consists of 49 items translated from the original English version and five items specific to the Japanese [17]. The summary score ranges from 0 to 216, with a lower score indicating better OHRQoL. If any of the OHIP-J item scores were missing, they were imputed using the median of the non-missing values for the patient. However, datasets with five or more missing responses were excluded from the analysis.

The daily energy intake (kcal) was calculated using the BDHQ, in which the participants reported the frequency of food and beverage intake in the previous month using a multiple-choice format.

Using the Eichner index, which evaluates the presence or absence of occlusal support from remaining teeth and fixed prostheses in four posterior zones (bilateral premolars and molars), participants were classified into two groups: those with at least one occlusal support zone and those without any occlusal support zones [18].

The quality of the dentures was evaluated by two trained dentists (R.K. and H.S.) based on stability and aesthetics [19]. Both factors were assessed using a 100-mm visual analog scale (VAS), with anchors of 0 indicating “very poor” and 100 indicating “very good.” The mean score was calculated to determine the patient’s overall denture quality [20]. For participants wearing dentures in both jaws, each was assessed separately, and the lower score was selected. The intraclass correlation coefficient (ICC) for inter-examiner reliability was 0.851 (95% confidence interval: 0.628-0.945), while the ICC for reproducibility was 0.921 (95% confidence interval: 0.764-0.975).

Masticatory performance was objectively evaluated using the gummy jelly method. Participants were instructed to chew a standardized gummy jelly (Glucolumn, GC Corp., Tokyo, Japan) for 20 seconds and then spit it out with 10 mL of water through a dedicated filter into a collection cup. The glucose concentration of the filtrate was measured using the Gluco Sensor GS-II (GC Corp.). In this study, the resulting glucose concentration was stratified in 50 mg/dL increments to assign a masticatory performance score (e.g., 0-50 mg/dL = 1, 51-100 mg/dL = 2), based on the literature [21].

CONUT score

Blood tests were performed to calculate the CONUT score, which is based on serum albumin levels, peripheral blood lymphocyte counts, and total cholesterol levels [14].

Participants were instructed to fast for eight hours before the blood draw, during which only water was permitted. Blood samples were collected in the morning and analyzed by BML, Inc. (Tokyo, Japan). The CONUT score offers a rapid and simple method for assessing malnutrition severity, particularly in inpatient settings, and is rated on a scale of 0 to 12. In order to identify individuals with low risk and those potentially at risk of malnutrition, the patients in this study were categorized into three groups based on previously published criteria [22]: low-risk (CONUT = 0), mild-risk (CONUT = 1 or 2), and moderate-risk (CONUT = 3 or 4), considering that the study population consisted of outpatients with few comorbidities and that the CONUT score ranges from a minimum of 0 to a maximum of 4.

Statistical analyses

The characteristics of the participants were compared between males and females using the Mann-Whitney U test or the chi-square test and among the three malnutrition risk groups using the Kruskal-Wallis test or the chi-square test. To investigate the factors associated with malnutrition risk as indicated by the CONUT score, the risk group was treated as the dependent variable (low-risk = 0; mild-risk = 1; and moderate-risk = 2), while the following factors were considered as independent variables: age, sex, BMI, CCI, nutrition-related media consumption, attention to a healthy diet, financial comfort, highest level of education, living arrangements, OHIP-J summary score, energy intake, Eichner index, denture quality score, and masticatory performance score. All statistical analyses were performed using IBM SPSS Statistics version 29 (IBM Corporation, Armonk, NY, USA) with a significance level of 0.05.

## Results

A total of 200 patients consented to participate; however, eight withdrew due to personal reasons, refusal of blood sampling, poor health, failure to attend, and refusal to complete the questionnaire. Thus, 192 participants were included in the analysis (mean age: 72.7 ± 8.4 years, 73 males and 119 females). The number of participants in the low-risk, mild-risk, and moderate-risk groups based on the CONUT score was 67, 116, and 9, respectively.

Males had significantly higher BMI, energy intake, and glucose concentration, while females had significantly higher OHIP-J summary scores (Table 1). Males were also significantly more likely than females to have a CCI score of 1 or higher, consume nutrition-related media less frequently, and live with family members.

**Table 1:** Differences in patient characteristics between males and females *P < 0.05. BMI: body mass index; OHIP-J: Japanese version of the Oral Health Impact Profile; VAS: visual analog scale; CONUT: Controlling Nutritional Status. ^†^Medians (first quartile–third quartile) are presented. Mann-Whitney U tests were applied. ^‡^Numbers (percentages) are presented. Chi-square tests were applied.

	Males (N = 73)	Females (N = 119)	P-value
Age^†^	75 (71–79)	73 (66–78)	0.139
BMI^†^	23.3 (21.1–25.4)	21.8 (19.5–24.9)	0.037*
Charlson comorbidity index^‡^			
0	37 (50.7%)	78 (65.6%)	0.041*
1–8	36 (49.3%)	41 (34.5%)	
Nutrition-related media consumption^‡^			
High	18 (24.7%)	54 (45.4%)	0.004*
Low	55 (75.3%)	65 (54.6%)	
Attention to a healthy diet^‡^			
High	61 (83.6%)	110 (92.4%)	0.056
Low	12 (16.4%)	9 (7.6%)	
Financial comfort^‡^			
High	54 (74.0%)	85 (71.4%)	0.702
Low	19 (28.6%)	34 (28.6%)	
Highest level of education^‡^			
Junior high or high school	39 (32.8%)	80 (67.2%)	0.561
Others (college, university, or higher)	21 (28.8%)	52 (71.2%)	
Living arrangements^‡^			
Living alone	10 (13.7%)	40 (33.6%)	0.002*
Living with family members	63 (86.3%)	79 (66.4%)	
OHIP-J summary score^†^	39 (23–58)	52 (30–66)	0.004*
Energy intake (kcal)^†^	2062 (1683–2441)	1651 (1345–2059)	< 0.001*
Eichner index^‡^			
With at least one occlusal support zone	44 (60.3%)	67 (56.3%)	0.589
Without any occlusal support zones	29 (39.7%)	52 (43.7%)	
Denture quality VAS score^†^	72.0 (63.3–82.3)	78.5 (65.0–85.0)	0.102
Glucose concentration (mg/dL)^†^	169 (139–195)	145 (119–193)	0.044*
Malnutrition risk level^‡^			
Low-risk (CONUT: 0)	16 (21.9%)	51 (42.9%)	
Mild-risk (CONUT: 1, 2)	53 (72.6%)	63 (52.9%)	0.013*
Moderate-risk (CONUT: 3, 4)	4 (5.5%)	5 (4.2%)	

For comparison across the malnutrition risk groups, significant differences were found for sex and BMI, and no significant differences were found for the other items (Table 2).

**Table 2:** Differences in patient characteristics among the malnutrition risk groups *P < 0.05. BMI: body mass index; OHIP-J: Japanese version of the Oral Health Impact Profile. ^†^Medians (first quartile–third quartile) are presented. Mann-Whitney U tests were applied. ^‡^Numbers (percentages) are presented. Chi-square tests were applied. ^a^Post hoc tests showed significant differences between the low-risk and mild-risk groups among both males and females (P < 0.05). ^b^Post hoc tests showed that the moderate-risk group had a significantly lower BMI than the low-risk group (P = 0.039).

	Low-risk (CONUT: 0)	Mild-risk (CONUT: 1, 2)	Moderate-risk (CONUT: 3, 4)	P-value
Age^†^	74 (65–78)	74 (70–78)	79 (73–82)	0.130
Sex^‡^				
Male	16 (23.9%)	53 (45.7%)	4 (44.4%)	0.013*^a^
Female	51 (76.1%)	63 (54.3%)	5 (55.6%)	
BMI^†^	23.2 (20.9–26.2)	22.3 (19.5–24.8)	18.4 (17.4–21.3)	0.001*^b^
Charlson comorbidity index^‡^				
0	46 (68.7%)	65 (56.0%)	4 (44.4%)	0.153
1–8	21 (31.3%)	51 (44.8%)	5 (55.6%)	
Nutrition-related media consumption^‡^				
High	30 (44.8%)	40 (34.5%)	2 (22.2%)	0.239
Low	37 (55.2%)	76 (65.5%)	7 (77.8%)	
Attention to a healthy diet^‡^				
High	59 (88.1%)	105 (90.5%)	7 (77.8%)	0.473
Low	8 (11.9%)	11 (9.5%)	2 (22.2%)	
Financial comfort^‡^				
High	48 (71.6%)	84 (72.4%)	7 (77.8%)	0.928
Low	19 (28.4%)	32 (27.6%)	2 (22.2%)	
Highest level of education^‡^				
Junior high or high school	22 (32.4%)	34 (29.3%)	4 (44.4%)	0.603
Others (college, university, or higher)	45 (67.2%)	82 (70.7%)	5 (55.6%)	
Living arrangements^‡^				
Living alone	21 (31.3%)	27 (23.3%)	2 (22.2%)	0.471
Living with family members	46 (68.7%)	89 (76.7%)	7 (77.8%)	
OHIP-J summary score^†^	44 (23–63)	46 (27–65)	41 (12–61)	0.640
Energy intake (kcal)^†^	1755 (1515–2292)	1820 (1463–2339)	1664 (1088–2014)	0.391
Eichner index^‡^				
With at least one occlusal support zone	36 (53.7%)	72 (62.1%)	3 (33.3%)	0.171
Without any occlusal support zones	31 (46.3%)	44 (37.9%)	6 (66.7%)	
Denture quality VAS score^†^	78 (63.0–84.5)	76 (65.0–83.9)	74 (50.8–85.3)	0.811
Glucose concentration (mg/dL)^†^	160 (124–201)	156 (121–190)	144 (84–179)	0.386

**Figure 1:**
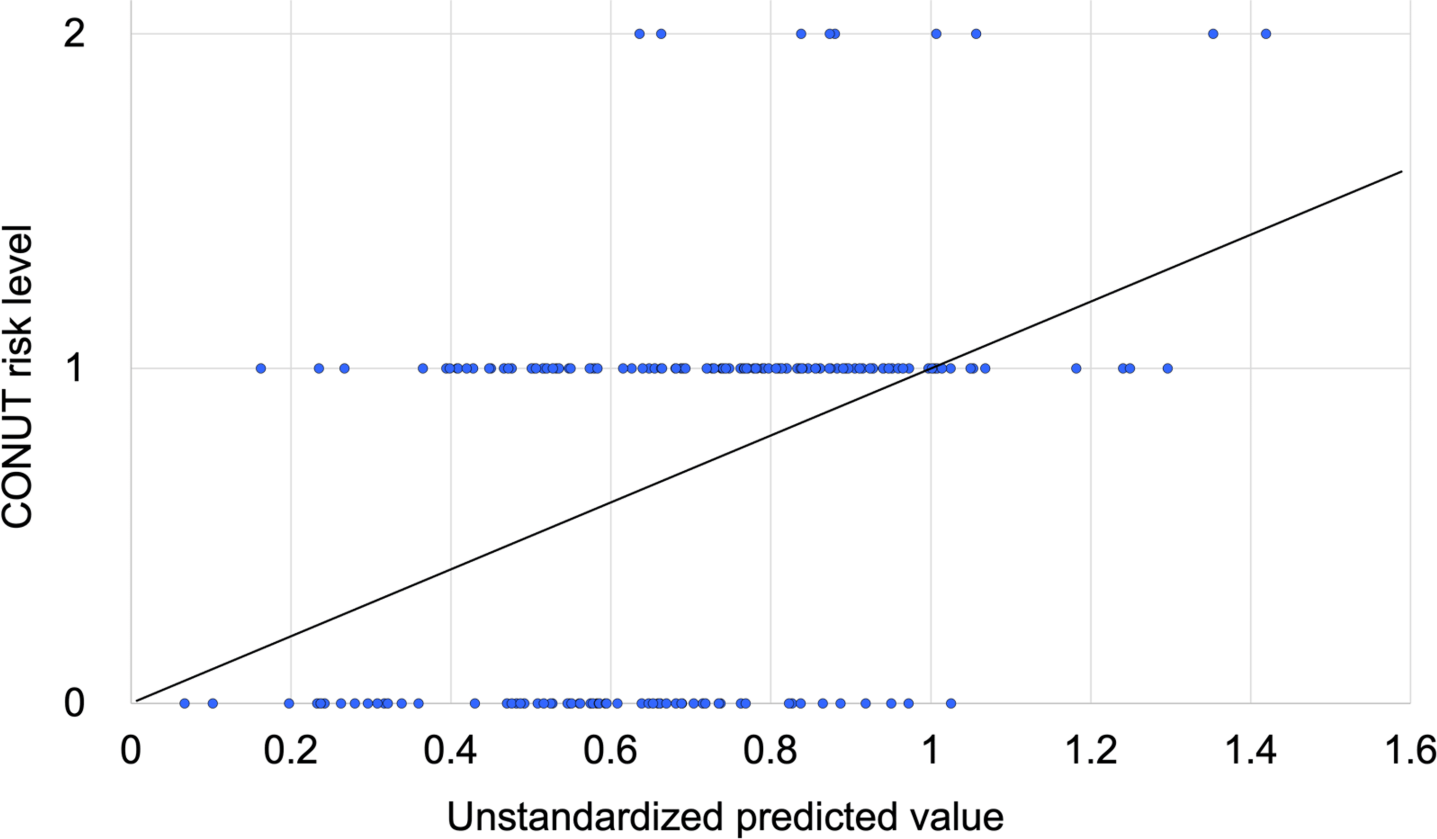
Scatter plot for the multiple regression model Regression model: *R*^2^ = 0.191; adjusted *R*^2^ = 0.127; *P* < 0.001. A multiple regression analysis was performed with the dependent variable (CONUT risk level) coded as 0 for low risk, 1 for mild risk, and 2 for moderate risk. Each point represents an individual participant, and the unstandardized predicted values based on the regression model were calculated using statistical software.

Multiple regression analysis revealed that males (*P* = 0.004), low BMI (*P* < 0.001), and low masticatory performance score (*P* = 0.025) were significantly associated with increased malnutrition risk as defined by the CONUT score (model fit: *R*^2^ = 0.191; adjusted *R*^2^ = 0.127; *P* < 0.001; Table 3 and Figure 1). However, OHIP-J summary score, denture quality VAS score, social background, and Eichner index were not significantly associated with malnutrition risk.

**Table 3:** Results of the multiple regression analysis *P < 0.05. SE: standard error; CI: confidence interval; BMI: body mass index; OHIP-J: Japanese version of the Oral Health Impact Profile; VIF: variance inflation factor. Regression model fit: *R*^2^ = 0.191; adjusted *R*^2^ = 0.127; *P* < 0.001. A multiple regression analysis was performed with the dependent variable coded as 0 for low risk, 1 for mild risk, and 2 for moderate risk. Multicollinearity is not a concern due to the low VIF values. ^†^Glucose concentration was stratified in 50 mg/dL increments to assign a masticatory performance score.

	Unstandardized coefficients		Standardized coefficients	95%CI	P-value	VIF
	Β	SE	β			
Age	0.005	0.005	0.078	−0.005 to 0.015	0.310	1.297
Sex (male)	0.261	0.089	0.230	0.085 to 0.437	0.004*	1.350
BMI	−0.044	0.011	−0.278	−0.066 to −0.022	<0.001*	1.093
Charlson comorbidity index (1–8)	0.101	0.080	0.090	−0.057 to 0.260	0.210	1.123
Nutrition-related media consumption (high)	−0.098	0.085	−0.086	−0.265 to 0.069	0.249	1.204
Attention to a healthy diet (high)	−0.005	0.131	−0.003	−0.263 to 0.253	0.969	1.195
Financial comfort (high)	0.011	0.090	0.009	−0.167 to 0.188	0.906	1.157
Highest level of education (junior high or high school)	−0.010	0.086	−0.009	−0.179 to 0.158	0.904	1.128
Living arrangements (living with family members)	0.075	0.090	0.059	−0.103 to 0.254	0.408	1.126
OHIP-Jsummary score	0.001	0.001	0.039	−0.002 to 0.004	0.594	1.189
Energy intake	0.000	0.000	−0.114	0.000 to 0.000	0.137	1.270
Eichner index (with at least one occlusal support zone)	0.071	0.083	0.064	−0.09 to 0.235	0.391	1.210
Denture quality VAS score	0.000	0.003	0.007	−0.006 to 0.006	0.923	1.237
Masticatory performance score^†^	−0.078	0.034	−0.162	−0.145 to −0.010	0.025*	1.123

## Discussion

This study explored the factors associated with objective nutritional status, as measured by the CONUT score, in patients with removable dentures. Multiple regression analysis showed that male sex, low BMI, and low masticatory performance score were significantly associated with increased malnutrition risk as defined by the CONUT score. Consequently, the null hypothesis was rejected. Among the variables examined, low BMI (standardized coefficient: −0.28) showed the strongest association with increased malnutrition risk, followed closely by male sex (standardized coefficient: 0.23). Additionally, patients with impaired masticatory performance were more likely to be at higher risk of malnutrition (standardized coefficient: −0.16). These findings suggest that maintaining and improving masticatory function and preventing a decline in BMI may positively influence nutritional status as measured by the CONUT score in denture wearers.

This study utilized the CONUT score, a screening tool that provides an objective and straightforward assessment of malnutrition based on three blood test parameters. It is commonly employed in the medical field for patients at high risk of malnutrition who require hospitalization or surgical procedures [23]. While the GLIM criteria are currently recommended for assessing nutritional status in adults [11], they classify malnutrition severity into only two levels (moderate and severe), making it difficult to identify individuals at mild risk for malnutrition, as was the case with the participants in this study. Moreover, the GLIM criteria require a validated nutritional screening tool, such as the MNA short form. Although the MNA short form is noninvasive, its application is limited to individuals aged 65 years or older who can self-report. In contrast, the CONUT score provides an objective and rapid assessment of the nutritional status for adults of any age. With a wide scoring range of 0 to 12, it is capable of detecting mild, moderate, and severe malnutrition, making it effective for continuous nutritional monitoring and management [14]. Additionally, the CONUT score is valuable for retrospective studies where blood test results are available. While one study reported the application of the CONUT to outpatients with type 2 diabetes [24], no study has applied the CONUT in the dental field for outpatients. The CONUT scores between 2 and 4 generally indicate mild malnutrition. In this study, the distribution of CONUT scores among participants was as follows: 67 patients scored 0, 66 scored 1, 50 scored 2, 5 scored 3, and 4 scored 4, with approximately 30% of the participants categorized as having mild malnutrition. Considering the relatively small number of undernourished patients in the study population and the focus on identifying individuals at low, mild, and moderate risk of malnutrition, this study employed a three-category classification: low-risk (CONUT: 0), mild-risk (CONUT: 1, 2), and moderate-risk (CONUT: 3, 4), as outlined in a previous study [22].

Regarding the association between masticatory function and nutritional status, it has been reported that masticatory performance, as evaluated by gum chewing, was significantly associated with nutritional status as measured by serum albumin levels [9]. Additionally, a cross-sectional study of Japanese community-dwelling older adults reported that oral frailty, which includes decreased masticatory performance, was associated with poor nutritional status as measured by serum albumin levels [25]. Although the nutritional indicators used differ, the association found between malnutrition and impaired masticatory performance in this study generally agrees with previous studies.

This study found that the risk of malnutrition was higher among men. Based on the questionnaire responses in this study, females were more likely to pay attention to a healthy diet compared to males (92.4% of females vs. 83.6% of males) and were also more likely to frequently check nutrition-related media than males (45.4% of females vs. 24.7% of males). These trends are consistent with previous studies indicating that older females are more likely to engage in health-related behaviors than males, such as following a healthy diet [26]. Overall, it is likely that increased health awareness and related behaviors of the current participants, especially among females, contributed to their better nutritional status [27]. In addition, it is possible that dietary habits, such as a lack of dietary diversity, and social challenges, such as social isolation, may have contributed to the finding that being male was associated with the increased risk of malnutrition. However, these factors were not examined within the scope of this study. Further research is needed to address these predictors.

In this study, no significant association was found between nutritional status and OHRQoL, as indicated by OHIP-J scores, or denture quality assessed by dentists. The OHIP is a comprehensive OHRQoL assessment tool based on responses from the past month. Allen [27] compared pre- and post-treatment outcomes of new dentures in older edentulous patients at a university hospital and reported that food choices remained similar before and after treatment, with no correlation between nutritional assessment using the MNA and OHRQoL measured by the OHIP. Although the study design was different, the current study results are generally consistent with those of the study by Allen [27].

The present study did not find an association between nutritional status and the Eichner index evaluating the presence of occlusal support. A previous study reported an association between the Eichner index and the CONUT score in patients with gastric cancer [28]; however, 60.5% of patients who participated in the study did not wear removable dentures. In the study, all participants had been using removable dentures for more than one month and maintained 22-28 functional teeth. Therefore, participants without occlusal supports may have compensated for their impaired masticatory function and mitigated the impact of loss of occlusal supports on their nutritional status by wearing dentures. Indeed, it has been reported that in edentulous patients who do not wear dentures, changes in dietary habits due to a decline in masticatory function may affect their nutritional status [29].

This study has certain limitations. First, the study participants may have included patients with pre-frailty and oral frailty. In literature, frailty and oral frailty have been reported to be positively associated with a decline in nutritional status, as measured by the MNA [25,30]. Additionally, detailed dietary habits, including dietary diversity, maybe a potential unmeasured confounder, and there remains the possibility of residual confoundings, such as socioeconomic factors, given the observational nature of this study. These factors may have influenced the nutritional status. Further research incorporating these parameters is warranted to provide a more comprehensive understanding. Second, as this study was conducted in a university clinic, caution should be exercised when generalizing the results to the broader population. Third, because this was a cross-sectional study, the associations identified cannot establish causality. The factors significantly associated with better nutritional status, such as better masticatory performance and female sex, should be further investigated. Since a better masticatory function can be achieved by prosthetic treatment [9] and females are more concerned about a healthy diet [26], future prospective studies with interventions such as prosthetic treatment or nutritional guidance are needed to investigate their impact on nutritional status.

## Conclusions

This is the first study in the dental field to examine factors associated with nutritional status using the CONUT score in patients with removable dentures and to quantify the magnitude of these associations. Regression analysis identified that male sex, low BMI, and impaired masticatory performance were significantly associated with malnutrition risk levels, as measured by the CONUT score. These findings highlight the association between nutritional status, as assessed through blood test results, and masticatory function in removable denture users, underscoring the importance of oral health in maintaining and improving nutritional status. The results of this study provide foundational information for future confirmatory prospective interventional studies.

## References

[1] Kosaka T, Kida M, Kikui M (2018). Factors influencing the changes in masticatory performance: the Suita study. JDR Clin Trans Res.

[2] Tôrres AC, Maciel AQ, de Farias DB, de Medeiros AK, Vieira FP, Carreiro AD (2019). Technical quality of complete dentures: influence on masticatory efficiency and quality of life. J Prosthodont.

[3] Watanabe H, Abe Y, Kusumoto Y (2024). Effect of treatment with implant-supported fixed partial dentures on oral health-related quality of life in patients with unilateral shortened dental arch. J Dent Sci.

[4] Ishimiya M, Nakamura H, Kobayashi Y (2018). Tooth loss-related dietary patterns and cognitive impairment in an elderly Japanese population: the Nakajima study. PLoS One.

[5] Horibe Y, Ueda T, Watanabe Y (2018). A 2-year longitudinal study of the relationship between masticatory function and progression to frailty or pre-frailty among community-dwelling Japanese aged 65 and older. J Oral Rehabil.

[6] Takahashi S, Naganuma T, Kurita N (2023). Social isolation/loneliness and tooth loss in community-dwelling older adults: the Sukagawa study. Innov Aging.

[7] McKenna G, Allen PF, Flynn A, O'Mahony D, DaMata C, Cronin M, Woods N (2012). Impact of tooth replacement strategies on the nutritional status of partially-dentate elders. Gerodontology.

[8] Madhuri S, Hegde SS, Ravi S, Deepti A, Simpy M (2014). Comparison of chewing ability, oral health related quality of life and nutritional status before and after insertion of complete denture amongst edentulous patients in a Dental College of Pune. Ethiop J Health Sci.

[9] Motokawa K, Mikami Y, Shirobe M (2021). Relationship between chewing ability and nutritional status in Japanese older adults: a cross-sectional study. Int J Environ Res Public Health.

[10] Kawashima Bori F, Fukuhara M, Masaki C (2020). The relationship between masticatory performance and intakes of foods and nutrients in Japanese male workers: a cross-sectional study. J Oral Rehabil.

[11] Cederholm T, Jensen GL, Correia MI (2019). GLIM criteria for the diagnosis of malnutrition - a consensus report from the global clinical nutrition community. Clin Nutr.

[12] Nakamura M, Ojima T, Nagahata T (2019). Having few remaining teeth is associated with a low nutrient intake and low serum albumin levels in middle-aged and older Japanese individuals: findings from the NIPPON DATA2010. Environ Health Prev Med.

[13] Taylor BE, McClave SA, Martindale RG (2016). Guidelines for the provision and assessment of nutrition support therapy in the adult critically ill patient: Society of Critical Care Medicine (SCCM) and American Society for Parenteral and Enteral Nutrition (A.S.P.E.N.). Crit Care Med.

[14] Ignacio de Ulíbarri J, González-Madroño A, de Villar NG (2005). CONUT: a tool for controlling nutritional status. First validation in a hospital population. Nutr Hosp.

[15] Green SB (1991). How many subjects does it take to do a regression analysis. Multivariate Behav Res.

[16] Charlson ME, Pompei P, Ales KL, MacKenzie CR (1987). A new method of classifying prognostic comorbidity in longitudinal studies: development and validation. J Chronic Dis.

[17] Yamazaki M, Inukai M, Baba K, John MT (2007). Japanese version of the Oral Health Impact Profile (OHIP-J). J Oral Rehabil.

[18] Kikutani T, Tamura F, Nishiwaki K (2009). Oral motor function and masticatory performance in the community-dwelling elderly. Odontology.

[19] Inukai M, Baba K, John MT, Igarashi Y (2008). Does removable partial denture quality affect individuals' oral health?. J Dent Res.

[20] Abe Y, Matsumoto T, Watanabe H, Gupta DK, Baba K (2021). Structural equation modeling for factors influencing patients' willingness to replace removable dentures. J Oral Sci.

[21] Hayashi Y, Fueki K, Yoshida-Kohno E, Inamochi Y, Wakabayashi N (2021). Responsiveness of methods to evaluate objective masticatory function in removable partial denture treatments. J Prosthodont Res.

[22] Tsuda S, Nakayama M, Tanaka S (2023). The association of controlling nutritional status score and prognostic nutritional index with cardiovascular diseases: the Fukuoka Kidney disease Registry study. J Atheroscler Thromb.

[23] Hayashi T, Fujiwara Y, Masuda M (2023). Time course and characteristics of the nutritional conditions in acute traumatic cervical spinal cord injury. Spine Surg Relat Res.

[24] Shiroma K, Tanabe H, Takiguchi Y (2023). A nutritional assessment tool, GNRI, predicts sarcopenia and its components in type 2 diabetes mellitus: a Japanese cross-sectional study. Front Nutr.

[25] Iwasaki M, Motokawa K, Watanabe Y (2020). Association between oral frailty and nutritional status among community-dwelling older adults: the Takashimadaira Study. J Nutr Health Aging.

[26] Cai L, Zhang L, Liu X (2024). Empirical analysis of health-related behaviors among older Hakka adults: a latent class analysis. Front Public Health.

[27] Allen PF (2005). Association between diet, social resources and oral health related quality of life in edentulous patients. J Oral Rehabil.

[28] Abe A, Ito Y, Hayashi H (2022). Relationship between nutritional biomarkers and occlusal status in gastric cancer patients using the Eichner index: observational study. Medicine (Baltimore).

[29] Lamy M, Mojon P, Kalykakis G, Legrand R, Butz-Jorgensen E (1999). Oral status and nutrition in the institutionalized elderly. J Dent.

[30] Soysal P, Isik AT, Arik F, Kalan U, Eyvaz A, Veronese N (2019). Validity of the mini-nutritional assessment scale for evaluating frailty status in older adults. J Am Med Dir Assoc.

